# Retroviruses of Bats: a Threat Waiting in the Wings?

**DOI:** 10.1128/mBio.01941-21

**Published:** 2021-09-07

**Authors:** Joshua A. Hayward, Gilda Tachedjian

**Affiliations:** a Health Security Program, Life Sciences Discipline, Burnet Institute, Melbourne, VIC, Australia; b Department of Microbiology, Monash University, Clayton, VIC, Australia; c Department of Microbiology and Immunology, The Peter Doherty Institute for Infection and Immunity, The University of Melbourne, Melbourne, VIC, Australia; Albert Einstein College of Medicine

**Keywords:** bats, virus, retrovirus, zoonosis, endogenous, restriction factors, KoRV

## Abstract

Bats are infamous reservoirs of deadly human viruses. While retroviruses, such as the human immunodeficiency virus (HIV), are among the most significant of virus families that have jumped from animals into humans, whether bat retroviruses have the potential to infect and cause disease in humans remains unknown. Recent reports of retroviruses circulating in bat populations builds on two decades of research describing the fossil records of retroviral sequences in bat genomes and of viral metagenomes extracted from bat samples. The impact of the global COVID-19 pandemic demands that we pay closer attention to viruses hosted by bats and their potential as a zoonotic threat. Here we review current knowledge of bat retroviruses and explore the question of whether they represent a threat to humans.

## INTRODUCTION

In the midst of the global coronavirus disease 2019 (COVID-19) pandemic, the role of bats as important hosts of zoonotic viral pathogens is difficult to dispute (1–4). Zoonotic viruses transmitted from bats include the Ebola virus and the severe acute respiratory syndrome (SARS) coronaviruses, which have had devastating impacts on individual and global scales (5–7). Although retroviruses have had a major and global impact on human health, the zoonotic potential of bat retroviruses is unknown at this time.

The family *Retroviridae* includes the human immunodeficiency virus (HIV) and human T-lymphotropic virus (HTLV), both of which are zoonotic viruses, that spilled over into humans from nonhuman primates (8, 9). HIV (which includes HIV type 1 [HIV-1] and HIV type 2 [HIV-2]) has infected 77.5 million people with 34.7 million attributed deaths since the start of the AIDS pandemic (10). HTLV type I (HTLV-1) currently infects an estimated 5 to 10 million people (11) and causes morbidity or mortality in 5 to 10% of infected individuals (12). On evolutionary timescales, members of the retrovirus family have been extensively transmitted between highly divergent animal species with evidence of past retroviral infections ubiquitously present across metazoan genomes (13–15).

Circulating infectious bat retroviruses have only recently been reported (16, 17). This is surprising given that modern retroviruses are known to infect a wide range of vertebrates and that bats (order Chiroptera) collectively represent over 20% of all mammalian species (18). Regardless, our knowledge about bat retroviruses has been growing steadily over the last two decades from studying retroviral sequences within bat genomes that are present due to key features of the retroviral replication cycle (19). These features are the synthesis of a complementary DNA (cDNA) of the viral RNA genome and integration of this cDNA into the host genome, establishing a provirus. Through infected germ line cells, these proviruses occasionally become fixed in the host species’ gene pool, giving rise to “endogenous” retroviruses (ERVs) (13, 20). ERVs representing ancient infectious retroviruses within the host genome accumulate mutations over the course of evolutionary history (13). These ERVs eventually become defective, fragmented, and incapable of producing viral particles or proteins (20, 21). Gene pool fixation has occurred throughout evolutionary history to the extent that, for example, approximately 8% of the human genome is derived from ERVs (22). It is through the analysis of ERV genomic “fossils” that we have learned much about the history of retroviral infection in humans and other animals, including bats, and from which we can make inferences about the potential existence of currently circulating, infectious “exogenous” retroviruses (XRVs) that remain to be discovered.

The COVID-19 pandemic provides a renewed impetus to identify potential viral threats harbored by bats that might emerge in the future so that we can establish proactive approaches to diagnose, prevent, and treat infections. Here we review how bats have been infected by retroviruses throughout their evolutionary history and the recent discoveries of exogenous retroviruses currently circulating in bats. We consider how analysis of components of the bat innate immune system, which are known to target retroviruses in other mammals, can provide insights into the relationship between bats and retroviruses. We outline the challenges in determining whether retroviral sequences identified in host samples represent infectious exogenous retroviruses versus fossilized endogenous retroviruses. Finally, we review the ability of bats to receive and transmit retroviruses from other species and across physical barriers and biogeographical boundaries, as well as examine whether exogenous retroviruses hosted by bats represent potential threats to humans.

## BAT RETROVIRUSES: A “RETRO”-SPECTIVE IN BRIEF

The earliest descriptions of an association between bats and retroviruses were studies in the 1970s to 1980s with the TB 1 Lu cell line and its derivative, the BLV-bat_2_ cell line, generated from lung tissue of the Brazilian free-tailed bat (Tadarida brasiliensis, family Molossidae) (23). These studies revealed that bat cells are susceptible to infection by retroviruses from diverse mammalian species. These retroviruses include the baboon endogenous virus (BaEV) (24) and the Cas E number 1 murine leukemia virus (MLV) (25) from the *Gammaretrovirus* genus, as well as the bovine leukemia virus (BLV) (22, 23) from the *Deltaretrovirus* genus.

Classical virus discovery in bats (e.g., rabies virus) usually relies on culture-dependent methods (2). However, all discoveries of bat retroviruses have stemmed from the use of culture-independent methods, including PCR and high-throughput sequencing (HTS) technologies. The first report of a bat retrovirus sequence, published in 2004, described the presence of ERVs from the genus *Betaretrovirus* within rodent genomes (26). This betaretroviral sequence, Carollia perspicillata endogenous betaretrovirus 5 (CpERV-β5), was identified within the genome of Seba’s short-tailed bat (C. perspicillata, family Phyllostomidae). This discovery was made as the result of a homology search of publicly available mammalian HTS genome sequences, which included *C. perspicillata*, as part of the National Institutes of Health Intramural Sequencing Center’s (NISC) Comparative Vertebrate Sequencing Initiative (26). While CpERV-β5 represents an incomplete and defective ERV, it was the first reported evidence of a putative natural retroviral infection in the ancestors of modern bats (26). Some years later, in 2008, a defective bat ERV, Myotis lucifugus endogenous retrovirus (Mlu-ERV) from the *Gammaretrovirus* genus, was similarly identified in genome sequences of the little brown bat (M. lucifugus, family Vespertilionid) (27).

The widespread adoption of HTS technologies has empowered virologists to access the treasure trove of retroviral infection records locked in each species’ genome. For bat retroviruses, this came in 2012-2013 as genomic and transcriptomic analyses led to the identification of many and diverse endogenous gammaretroviruses and betaretroviruses across a range of different bat species from both bat suborders, the Yangochiroptera and Yinpterochiroptera (28–30). Since then, retroviral sequences have been reported for 51 species of bats (Table 1). Of particular note was the 2017 discovery of *Miniopterus* endogenous retrovirus (MINERVa) in the genomes of long-fingered bats (family Miniopteridae) (31), which was the first report of an endogenous deltaretrovirus in any animal.

**TABLE 1:**
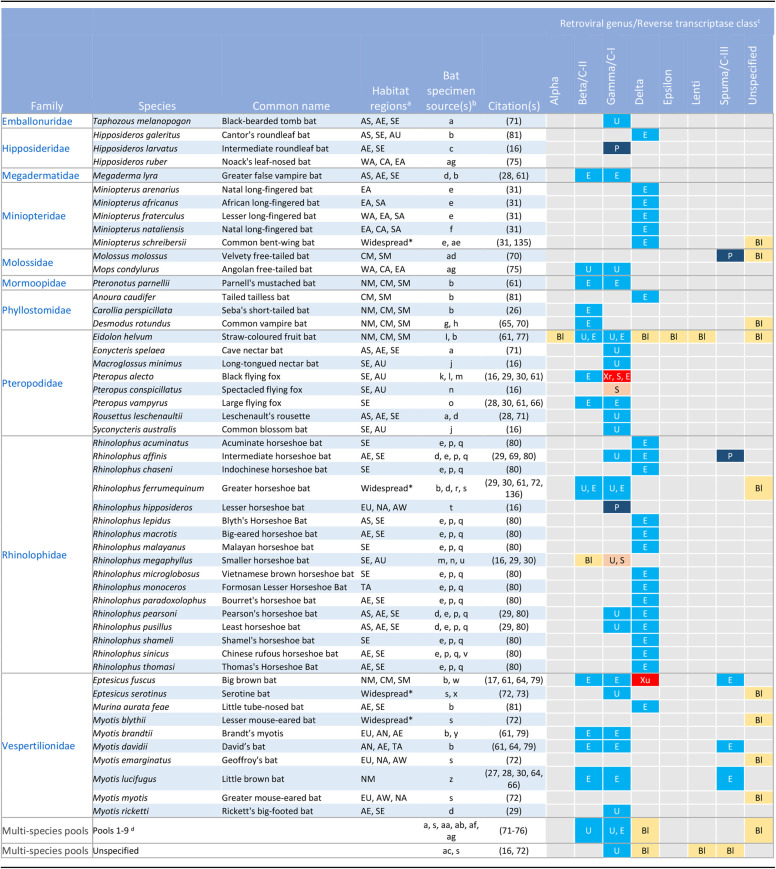
Reported retrovirus sequences in bats

a Bat habitat geographical regions as graphically delineated in Fig. 2. Abbreviations: AC, Central Asia; AE, Eastern Asia; AN, Northern Asia; AS, Southern Asia; AU, Australia; AW, Western Asia; CA, Central Africa; CM, Central America; EA, Eastern Africa; EU, Europe; NA, Northern Africa; NM, Northern America and the Caribbean; SA, Southern Africa; SE, South East Asia; SM, Southern America; TA, Taiwan; WA, Western Africa. Bats with habitats spanning four or more geographical regions across multiple continents that are denoted as widespread are indicated by asterisks.

b Bat specimen sources as reported by the adjacent citation(s): a, Yunnan, China; b, Not specified; c, Guangxi, China; d, China; e, National Museum, Czech Republic; f, De Hoop Nature Reserve, South Africa; g, Mexico; h, Berlin Zoological Gardens, Germany; i, Accra and Tano Sacred Grove, Ghana; j, Daintree Rainforest, Australia; k, Hervey Bay, Australia; l, Brisbane, Australia; m, Australia; n, Queensland, Australia; o, Lubee Bat Conservancy, Florida, USA; p, Charles University, Czech Republic; q, Hungarian Natural History Museum, Hungary; r, Gyeongju, Yeongju, Ulju, Sunchang, and Jindo, South Korea; s, Croatia; t, Sichuan, China; u, Booloumba Creek Caves, Australia; v, Anhui, China; w, South Dakota, USA; x, Sers, France; y, West Russia; z, Framingham, Massachusetts, USA; aa, Sedon site, Myanmar; ab, Wutao site, Myanmar; ac, Hervey Bay, Boonah, Byron Bay, Alstonville, Nambucca Heads, Australia; ad, Cacao and La Chaumière, French Guiana; ae, Cave Pionirska pećina, Mt. Beljanica, Serbia; af, Davao, Philippines; ag, multiple caves and forests, Guinea.

c Retroviral genera, indicated by their name-prefix or equivalent reverse transcriptase class (C-I, -II, and -III) reported for each species/pool. Letter codes indicate the nature of the reported retroviruses: Bl, reported homology to retroviral sequences by BLAST analysis; E, endogenous retrovirus(es) (ERV); P, unverified but potential exogenous retrovirus (XRV) as reported genomic sequence although incomplete is derived from ultracentrifuged viral pellets and replication competence is unknown; S, Bat(s) are seropositive to retroviral antigens; U, sequences of undetermined nature as methods used cannot substantially differentiate ERVs from XRVs; Xr, XRV with complete genome sequence reported and intact with verified replication competence; Xu, XRV although reported genome sequence is incomplete and replication competence remains to be verified.

d Pool 1, Eonycteris spelaea, Hipposideros armiger, Hipposideros cineraceus, Rousettus leschenaultii, Taphozous melanopogon; pool 2, H. armiger, Myotis chinensis, Rhinolophus ferrumequinum; pool 3, H. armiger, H. fulvus, Megaderma lyra, M. chinensis, R. ferrumequinum; pool 4, Miniopterus schreibersii, Myotis nattereri; pool 5, Cynopterus brachyotis, Macroglossus minimus, Ptenochirus jagori, R. amplexicaudatus, R. rufus; pool 6, Rhinolophus sp.; pool 7, Myotis bechsteinii, M. nattereri, Myotis myotis, Nyctalus leisleri, Barbastella barbastellus, Pipistrellus kuhlii; pool 8, Pipistrellus pipistrellus, Pipistrellus pygmaeus; pool 9, Myotis daubentonii, Vespertilio murinus.

In 2020, the first confirmed bat XRV, the Hervey pteropid gammaretrovirus (HPG), was reported, detected in the feces of the black flying fox (Pteropus alecto, family Pteropodidae) (16). HPG and several closely related gammaretroviruses were found, by serological and PCR analysis, to be circulating in populations of P. alecto, along with multiple other pteropid bat species in northeastern Australia (16). Later the same year, a second XRV, Eptesicus fuscus deltaretrovirus (EfDRV), was revealed to be circulating in big brown bats (E. fuscus, family Vespertilionidae) in North America by PCR analysis (17).

## BAT IMMUNOLOGY RESEARCH PROVIDES CLUES TO THE RELATIONSHIP BETWEEN BATS AND RETROVIRUSES

Other avenues of research have also provided insights into the relationship between bats and infection with retroviruses. The immune system of bats has become an area of great interest in recent years, as bats gained growing attention as reservoirs of human viral pathogens (18, 32–34). Restriction factors are proteins that directly target viral components and are at the vanguard of the innate immune response. The best studied of these proteins (e.g., APOBEC3G, tetherin, TRIM5, and MX2), were discovered and characterized through research on the retrovirus, HIV-1 (35–43).

Studies of bat restriction factors have yielded insights into the relationship between bats and retroviruses as their evolution in bats is likely driven by retroviral infection. The APOBEC3 family of proteins restricts retroviral replication through multiple mechanisms which can vary by individual APOBEC3 protein (44), and pteropid bats possess the largest and most diverse repertoire of *APOBEC3* genes of any mammal reported thus far (45). Furthermore, several bat APOBEC3 proteins are functional as determined by their ability to inhibit the replication of HIV-1 (45). Vesper bats are recently reported to harbor an expanded repertoire, compared to other mammals, of the restriction factor tetherin (46, 47). The pteropid orthologues of the human restriction factors, TRIM5α and MX2, inhibit the replication of gamma- and lentiviruses, respectively (48). Intriguingly, *P. alecto* possesses an unidentified restriction factor that inhibits the lentiviruses of primates but not those of other mammals (48). Collectively, these reports suggest that modern bats have an important and ongoing relationship with infectious retroviruses, many of which remain to be discovered.

## ERVs AND XRVs: THE CHALLENGE OF DETERMINING THE NATURE OF BAT RETROVIRAL SEQUENCES

While there are many reports of bat retrovirus sequences (Table 1), it is not always clear whether these sequences are derived from an ERV or an XRV due to challenges distinguishing between the two. First, integration of the retroviral DNA into the host genome is a standard aspect of the retroviral replication cycle, meaning that both ERV and XRV DNA may be detected in individual host samples. ERVs may be transcriptionally active, with expression regulated by the host (49, 50). Accordingly, both ERVs and XRVs can also be identified in host samples through their RNA. ERVs, including those that are defective, can potentially generate virus-like particles (VLPs) (51). These VLPs may be present in viral pellets and/or density fractions following ultracentrifugation of host samples. Nonviral particles, such as extracellular vesicles, can incorporate viral proteins and fragments of viral RNA. They can cosediment with virions during ultracentrifugation, causing difficulty distinguishing them from viral particles (52) unless appropriate purification strategies are employed (53). Last, even after endogenization, ERVs may remain capable of generating infectious viral particles that can be released and transmitted to other hosts, as observed for the koala retrovirus (KoRV) (54, 55). Under these circumstances, the retrovirus can behave as both a vertically transmitted ERV and a horizontally transmitted XRV.

It is because of the difficulty in distinguishing between ERVs and XRVs that so many bat retroviral sequences have been reported without being conclusively defined as an ERV or XRV (Table 1). This is often the case for broad viral metagenomic studies that analyze pools of nucleic acids extracted from host samples and report incidentally on the presence of retroviral contigs in the resultant HTS data sets. These difficulties have led at least one group to exclude retroviruses from a recent viral metagenomic analysis (56). We have summarized the main lines of evidence that may be provided in support of the existence of ERVs and XRVs (Table 2).

**TABLE 2:**
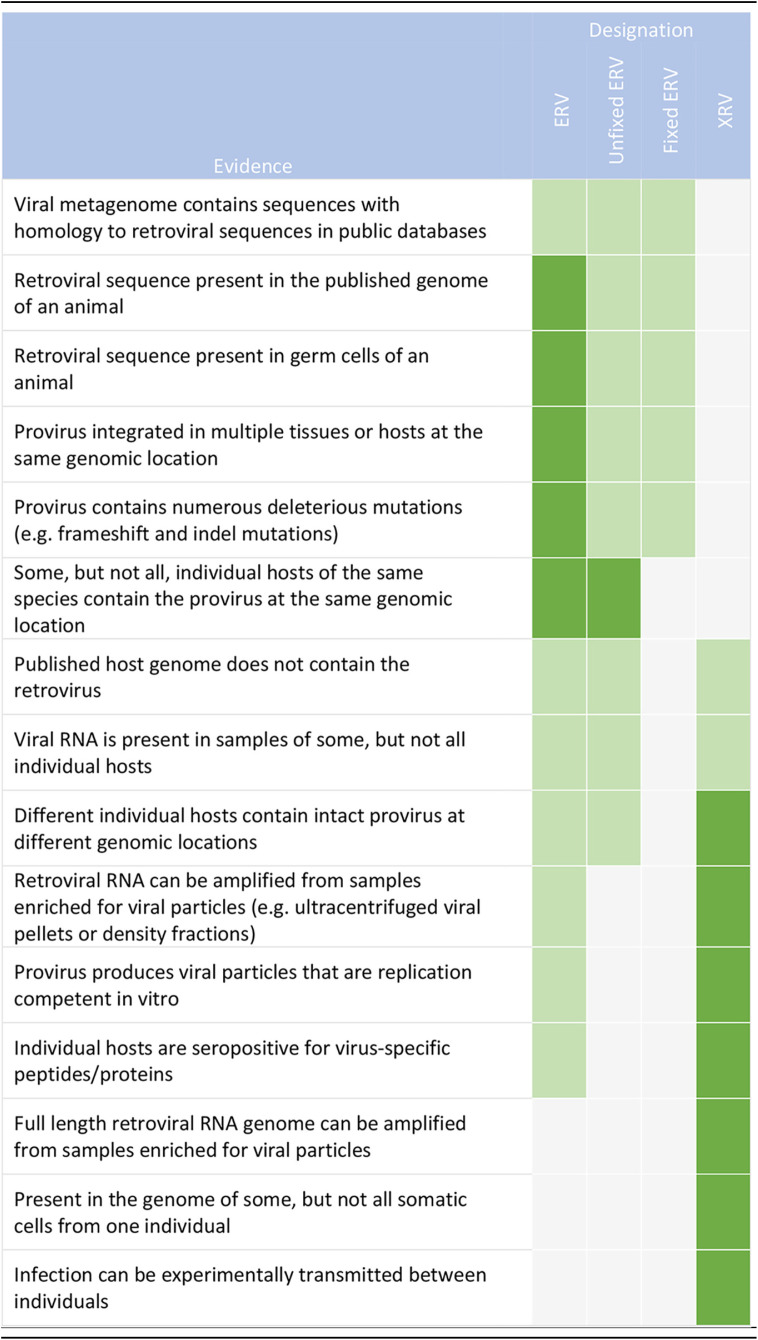
Evidence for differentiating endogenous and exogenous retroviruses in samples

a Strength of the evidence supporting the nature of any given retroviral sequence. Dark green indicates strongly supportive evidence; light green indicates moderately supportive evidence. ERV, endogenous retrovirus; XRV, exogenous retrovirus.

## THRESHOLDS OF EVIDENCE: ERVs

Knowing the challenges in differentiating between ERVs and XRVs allows the formulation of principles to generate a threshold of evidence to conclusively define them. ERVs are, by definition, endogenized in the germ line, and accordingly will be represented in the DNA of all tissues of the host and at the same genomic location. Where an analysis reveals that multiple tissues or individuals contain the same retrovirus at the same genomic location, it is an ERV. Importantly, where some but not all individuals of the host species contain a retroviral sequence at the same genomic location, it is likely an ERV that has not yet spread through the entire gene pool. This is the case for KoRV, which is presently undergoing endogenization in Australia’s koala populations (57–59). Conversely, where a retroviral sequence is represented in the genome of some but not all of the somatic cells of the host, this would likely be indicative of infection by an XRV.

ERVs are frequently ancient, and their sequences often contain deleterious mutations such as indels and frameshifts within the viral protein-coding sequences (13, 21). If a retroviral sequence is identified with many of these deleterious mutations, then it is clearly an ERV. Regardless, retroviral sequences with no obvious defects could still be either an ERV or XRV. This is because, as is the case with KoRV, an ERV that has only recently undergone endogenization may be intact and simply not present within the host genome for enough time to acquire deleterious mutations. Accordingly, further analyses are required to discern between ERVs and XRVs.

Often, when a species’ genome assembly contains an integrated retrovirus, it is reported as an ERV (Table 1). However, it is important to consider that genome assemblies are likely to be generated from, at most, a limited number of individual representatives of that species, and that ERVs identified in these assemblies may not necessarily be present throughout the gene pool of that species. This is the case for KoRV and some human ERVs (57, 60) and also appears to be the case for at least one bat ERV, Rhinolophus ferrumequinum retrovirus (RfRV), which is present in some, but not all members of its host species, the greater horseshoe bat (R. ferrumequinum, family Rhinolophidae) (61).

Many studies have reported the discovery of bat ERVs through analysis of published bat genomes by retroviral homology searches (e.g., BLAST) and/or the use of bioinformatic tools designed to identify ERVs (e.g., LTRharvest [62] or RetroTector [63]) (Table 1). This evidence is often supported through taxonomic classification by phylogenetic analysis, and additional identification of putative genes or sequence motifs characteristic of particular retroviral genera (28, 30, 64–66).

## THRESHOLDS OF EVIDENCE: XRVs.

In contrast to ERVs, the presence of XRVs are more difficult to confirm. Key characteristics of XRVs are the presence of full-length virion-associated retroviral RNA in samples enriched for viral particles and the production of replication competent virions with the capacity to be transmitted between individual hosts. The presence of retroviral RNA in host samples that is not reflected by homologous proviral DNA in the genomes of host germ cells, or across all other somatic cells, can additionally strengthen the case that the RNA is from an XRV rather than an ERV. Furthermore, demonstrating that the provirus is present at unique sites of integration in different individuals is a reflection of infection rather than endogenization.

To help establish that a retrovirus has the potential for transmission, researchers could first seek to demonstrate that a retrovirus is replication competent *in vitro*, i.e., that the proviral form of the virus is capable of generating viral particles that are able to enter new cells and establish a productive infection. In this regard, the complete retroviral genome sequence can be used to synthesize an infectious molecular clone, comprising the provirus, that can be transfected into cells to generate replication competent viral particles, as described for the bat retrovirus, HPG (16). Alternatively, classical culture-based virology could be used to isolate the virus from host samples and determine its replication competence (2).

To facilitate sequencing of the viral genome and/or the isolation of viral particles for culture-based analysis, various methods can be employed to enrich for viral particles, such as ultracentrifugation onto a sucrose cushion or through a continuous density gradient (67). However, retroviral particles are fragile, and care must be taken to recover them intact and infectious (68). The detection of retroviral RNA in ultracentrifuged material, while not conclusive on its own, could be considered preliminary evidence for the presence of viral particles and, potentially, an XRV.

The presence of a retroviral RNA with uninterrupted open reading frames derived from samples enriched for viral particles, demonstration that the virus is replication competent *in vitro*, and evidence of XRV transmission by either direct infection of the host or serological and/or nucleic acid (RNA) positivity in bat populations together provide a threshold of evidence for the identification of a novel XRV, which is met by recent reports to various extents (16, 17) (Table 1).

Two previous reports have described spumaviral sequences likely representing XRVs, but with insufficient evidence to confirm these observations (69, 70). Both of these reports were viral metagenomics studies, the first analyzing bats from Asia (69), the second analyzing bats from South America (70). Each of these studies described the identification of short spumaviral sequences derived from ultracentrifuged viral pellets. The spumaviruses described in these studies, Rhinolophus affinis foamy virus 1 (RaFV-1) and Molossus molossus foamy virus 1 (MomoFV-1), were discovered in the intermediate horseshoe bat (R. affinis, family Rhinolophidae) and the velvety free-tailed bat (M. molossus, family Molossidae), respectively (69, 70) (Table 1). RaFV-1 represents ∼2.8 kb covering part of the polymerase and envelope protein genes, while MomoFV-1 consists of a short 312 nucleotides (nt) sequence of the polymerase gene. No frameshift or indel mutations are present in the limited regions reported for the RaFV-1 and MomoFV-1 sequences (GenBank accession nos. JQ814855 and KX812444).

Another example of likely but unconfirmed XRVs are two gammaretroviral sequences, Hipposideros larvatus gammaretrovirus (HlGRV) and Rhinolophus hipposideros gammaretrovirus (RhGRV), in samples from the intermediate roundleaf bat (H. larvatus, family Hipposiderae) and the lesser horseshoe bat (R. hipposideros, family Rhinolophidae), respectively (both from Asia) (Table 1). These gammaretroviruses were reported alongside HPG and were assembled from public sequence read archives (SRA) generated from nucleic acids purified from a viral pellet (16, 69) (Table 1). HlGRV and RhGRV are incomplete assemblies covering all retroviral genes, and neither contains frameshift or indel mutations (GenBank accession nos. MN413613 and MN413614).

The most recent report of a bat XRV met the threshold of evidence required for the identification of an XRV (17). Hause et al. (17) derived the near complete genome sequence of EfDRV from a metagenomic analysis of homogenized lung and heart tissue samples of E. fuscus. The EfDRV sequence contains the genomic features of a deltaretrovirus and is phylogenetically closely related to recently reported endogenous bat deltaretroviruses and BLV (17, 31). EfDRV was not assessed for replication competence or other biological traits. However, quantitative reverse transcription-PCR (qRT-PCR) analysis of 60 individual E. fuscus bats revealed the presence of EfDRV RNA in 4 of 60 bats with 97 to 100% nucleotide identity to EfDRV. Importantly, EfDRV was absent from the published E. fuscus genome indicating that if it is an ERV, it is unfixed in the gene pool. This study concluded that EfDRV represents an XRV (17). The genome sequence of EfDRV was not derived from a viral pellet, and there is no direct evidence yet that it is capable of generating infectious viral particles. However, the key finding from this study was the detection of EfDRV RNA in some but not all individual bats. To strengthen this observation, the replication competence of EfDRV should be evaluated.

The discovery of the bat retrovirus, HPG, is supported by robust evidence (16). The complete intact genomic sequence of HPG was derived through metagenomic analysis of RNA from viral particles obtained from *P. alecto* bat feces. HPG particles were enriched by ultracentrifugation through a sucrose density gradient and amplified using a modified single-cell whole-transcriptome amplification procedure. The HPG sequence harbors the canonical *gag*, *pol*, and *env* genes as well as other typical genomic features of a gammaretrovirus, including a tRNA^Pro^ primer binding site. HPG is phylogenetically closely related to KoRV and the gibbon ape leukemia virus (GALV) with nucleotide identity of 74% and 78%, respectively. The HPG sequence was used to chemically synthesize a full-length infectious molecular clone, which generated viral particles following transfection of human 293T cells. The virions produced from these cells possess enzymatic and morphological traits consistent with a gammaretrovirus, including manganese-dependent reverse transcriptase activity and a spherical shaped core. These viral particles were capable of establishing successive rounds of infection in permissive cells, including human cells, *in vitro*. Recombinant HPG envelope protein was used to generate rabbit anti-HPG antibodies, establishing serological evidence of HPG and HPG-related viruses in Australian bats. Thirty-two percent of the bats tested were seropositive for HPG or HPG-related protein sequences. A qRT-PCR analysis of bat feces also revealed the presence of HPG-specific and HPG-related DNA and RNA. It is worth noting that the presence or absence of HPG nucleic acids was only directly assessed in bat feces, accordingly details regarding HPG’s residence within the blood or specific organs within bats is unknown at this time. Whether HPG replicates in bats at high or low titers is also unknown. In combination, the serological and nucleic acid data confirmed that HPG and HPG-related viruses are circulating in numerous individuals, across several species of Australian bats, in multiple locations across northeastern Australia. Further supporting this, the HPG sequence was not present in the published pteropid bat genomes, and the HPG sequence could not be amplified from genomic DNA purified from two separately collected *P. alecto* samples. Collectively, these data provide evidence that HPG is an infectious XRV that is not ubiquitously present across the gene pool of its host species (16).

Due to the ubiquity of endogenous retroviral sequences across metazoan genomes, the threshold of evidence provided in these studies (16, 17) is necessary to confirm the presence of XRVs. In the absence of strong evidence, retroviral sequences should be assumed to be derived from ERVs until convincing data suggest otherwise.

## BAT RETROVIRUSES THROUGH TIME AND SPACE

With the variable nature of reported retroviral sequences and the different types of evidence supporting these conclusions in mind, we compiled a summary of this body of literature. Table 1 summarizes all of the bat species for which retroviral sequences have been reported, the retroviral genera to which these sequences have been assigned, and the nature of these sequences insofar as they can be concluded from the presented evidence (Table 2). Information regarding the habitats of the bat species and the reported sources of the bat specimens is also included (Table 1).

**FIG 1:**
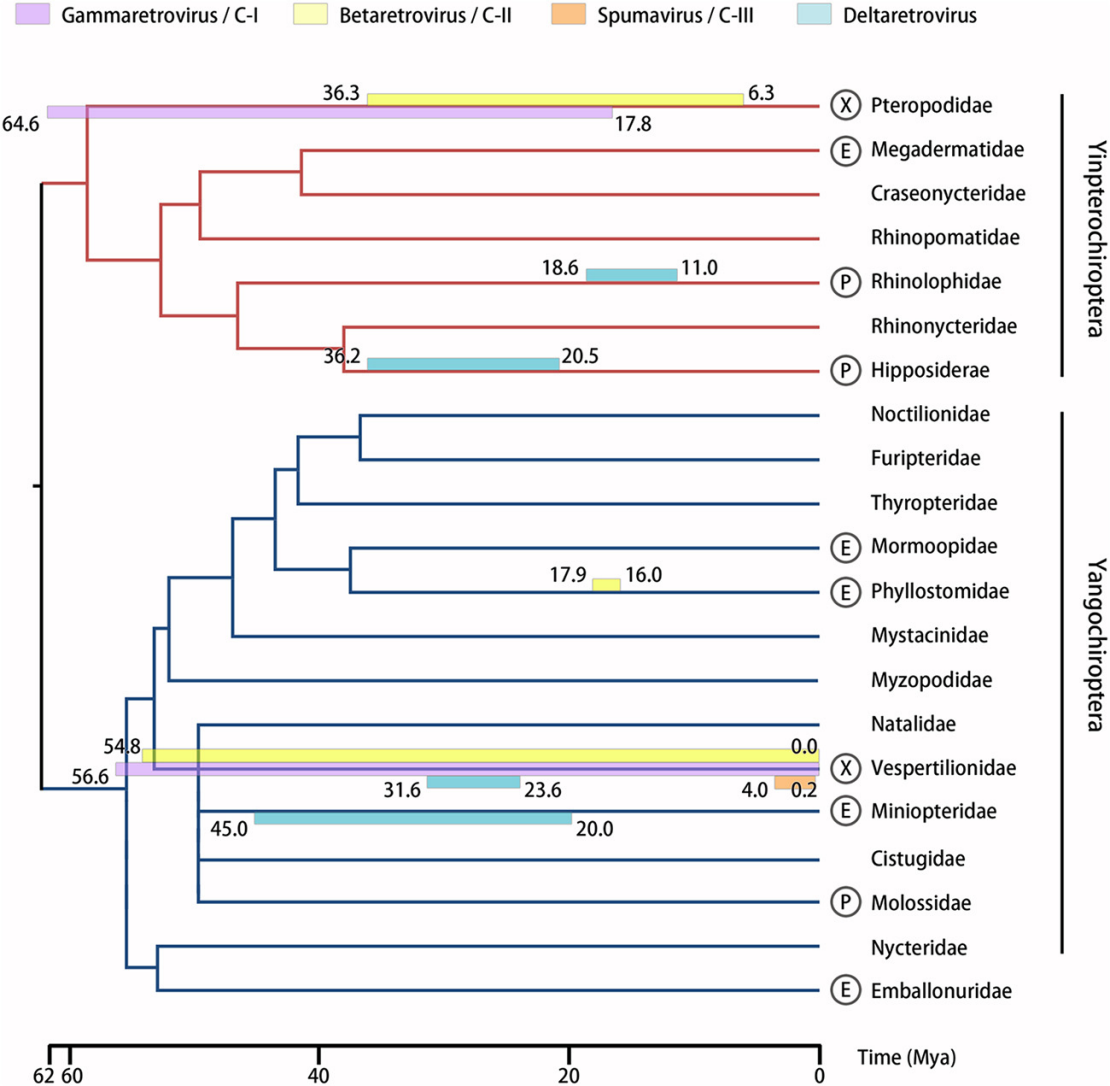
Integration of retroviruses into the genomes of bats. A phylogenetic tree representing divergence dates within the order Chiroptera was generated using TimeTree (134) and modified to include the recently recognized families, Miniopteridae and Cistugidae (18). Dates of reported integration events are indicated by colored bars overlapping the branches of bat families. Bar colors represent the retroviral genera/reverse transcriptase class (classes I to III) of the reported ERVs (see key). Letters next to the name of each bat family indicate the nature of reported retroviral sequences (see also Table 1): “X” indicates confirmed exogenous retroviruses (XRVs), “P” indicates potential but unconfirmed XRVs, and “E” indicates endogenous retroviruses (ERVs) or sequences of undetermined nature. The absence of a letter indicates that no sequences have been reported thus far.

**FIG 2:**
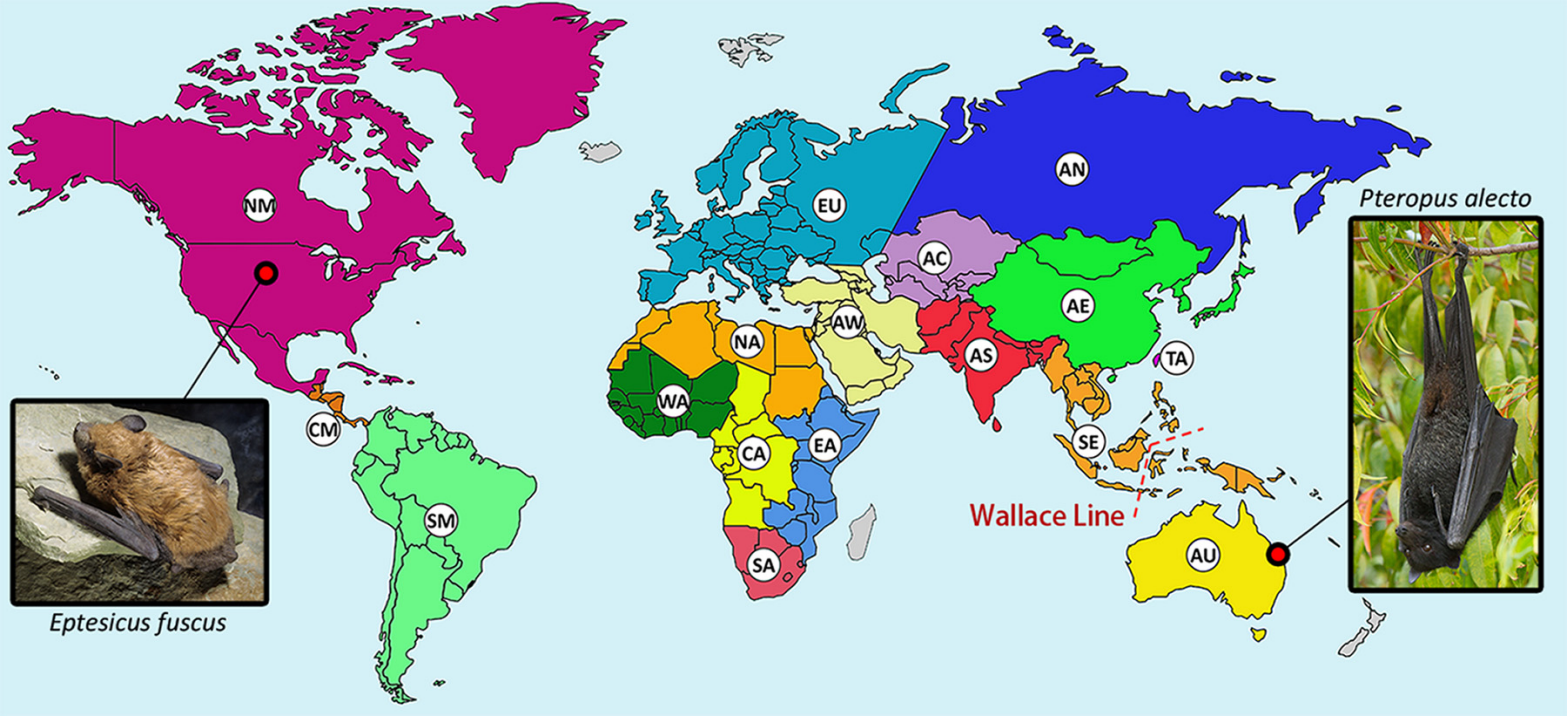
Distribution of bats from which retroviral sequences have been reported. Geographical regions hosting bat species with reported retroviral sequences are separated by color and labeled as follows: AC, Central Asia; AE, Eastern Asia; AN, Northern Asia; AS, Southern Asia; AU, Australia; AW, Western Asia; CA, Central Africa; CM, Central America; EA, Eastern Africa; EU, Europe; NA, Northern Africa; NM, Northern America and the Caribbean; SA, Southern Africa; SE, South East Asia; SM, Southern America; TA, Taiwan; WA, Western Africa. Regions in gray have not had reported bat retroviral sequences. A biogeographical boundary, the Wallace line, is indicated by the red dashed line. The hosts of two confirmed bat exogenous retroviruses (XRVs), the black flying fox (Pteropus alecto), and the big brown bat (Eptesicus fuscus) are pictured. “Black Flying Fox - Pteropus Alecto” by Andrew Mercer available at https://commons.wikimedia.org/wiki/File:Black_Flying_Fox_-_Pteropus_alecto_-_(IMG_4883).jpg under a Creative Commons Attribution-ShareAlike 4.0. “Big Brown Bat” by John MacGregor available at https://commons.wikimedia.org/wiki/File:Big_brown_bat_crawl.png under a Creative Commons Public Domain Mark 1.0.

Retroviral sequences have been reported for 51 bat species from 10 of 21 families within the order Chiroptera. Collectively, these bat species are distributed across all continents except Antarctica, and the reported endogenous bat retroviral sequences span ∼64 million years of evolutionary history (Table 1 and Fig. 1 and 2). A limitation of the compiled data is that in some studies, samples collected from different species were pooled prior to analysis, thereby introducing uncertainty regarding the specific origin of some bat retroviral sequences (16, 71–76). Bat species that are represented only in these multispecies pools are not included in the 51 listed species.

## DO BATS REALLY HOST ALL RETROVIRAL GENERA?

Sequences representing all retroviral genera (*Alpharetrovirus*, *Betaretrovirus*, *Gammaretrovirus*, *Deltaretrovirus*, *Epsilonretrovirus*, *Lentivirus*, and *Spumavirus*) have been reported to be associated with bats. However, the nature, extent, and confidence of these reports vary widely (Table 1), requiring caution in how these findings are interpreted.

The least robust evidence comes from viral metagenomic analyses which rely on simple BLAST homology of HTS contigs compared to retroviral sequences in public databases. For example, reports of bat alpharetroviruses, epsilonretroviruses, and lentiviruses have been identified only by using this strategy (72, 77). While there is value in the ease, speed, and scale of this approach, it is problematic if we want definitive identification of new retroviral sequences. The major limitations of this approach follow. (i) Contigs may only provide information about a limited region of the retroviral genome and retroviruses may undergo recombination, including between different genera, preventing accurate or confident identification based on short sequences (hundreds to low thousands of nucleotides). (ii) Public databases may not be carefully curated, and the reliability of homology matches should be treated with caution. (iii) If no close match exists in the database, then the best match by homology may be distantly, if at all, related. (iv) BLAST results are reported as probability scores (E values) of the matched sequences, and they are not an accurate or appropriate tool for taxonomic assignment. Reports relying on this approach should be interpreted with an understanding of its limitations. Specifically, with regard to alpharetroviruses, epsilonretroviruses, and lentiviruses (72, 77), more work is required to confirm their presence within bats.

The existence of bat betaretroviruses and gammaretroviruses has been extensively reported by multiple independent research groups (Table 1). Bat genomes are replete with endogenous beta- and gammaretroviruses. Reports detailing these ERVs include strong evidence of taxonomic identity supported by phylogenetic analysis of the evolutionary relatedness of retroviral sequences, as well as the description of genomic features characteristic of specific retroviral taxa (28–30, 61, 64–66, 71, 78, 79).

Bat spumaviruses (also described as foamy viruses) have been sporadically reported in the literature by several research groups (Table 1). In 2012, a viral metagenomic study identified a spumavirus sequence from a sample collected from the intermediate horseshoe bat (R. affinis, family Rhinolophidae) (69). Later, the genome of three vesper bats (family Vespertilionidae) was found to contain evolutionary evidence of recent integration by spumaviruses (64) (Fig. 1). These findings were supported by phylogenetic analyses (64, 69, 70).

More recent reports have described endogenous deltaretroviruses across the bat families Miniopteridae, Phyllostomidae, Hipposideridae, Rhinolophidae, and Vespertilionidae and the first identification of deltaretroviral fossil sequences in other diverse mammalian species, including meerkats, mongooses, dolphins, and solenodons (31, 80, 81). These reports are based on phylogenetic analyses and genomic characterizations. A report claiming to have identified deltaretroviral contigs generated from a pool of bat species representing multiple bat families had been reported previously (73). However, this claim was made solely on the basis of BLAST homology, and no deltaretroviral sequences were provided or deposited in public repositories for further analysis.

On the weight of the evidence, we can be confident that bats host, or have hosted, betaretroviruses, gammaretroviruses, deltaretroviruses, and spumaviruses. If bat retroviruses from other retroviral genera do exist, then robust evidence remains to be found.

## ENDOGENOUS BAT RETROVIRUSES REVEAL A LONG HISTORY OF INFECTION

Several studies focusing on endogenous bat retroviruses have analyzed the integration events from which various ERVs have originated (28, 30, 64, 65, 80, 81). Putative dates of integration for individual ERVs were estimated by analysis of the presence of mutations in the pairs of long terminal repeat (LTR) sequences in the integrated proviruses which, following integration, act as genetic paralogs mutating at the same rate as the host genome (28, 30, 64, 65). Alternatively, integration dates have been reported as an estimated range based on the presence or absence of the ERVs in closely related bat species coupled with their times of divergence (80, 81).

These reports allow us to start building a timeline of historical retroviral invasions of the genomes of bat families (Fig. 1). Some integrations are estimated to date back as far as 64.6 million years (mya) (28), and others as recently as modern times, i.e., within the range of thousands of years in cases where zero mutations are observed between paired LTRs (64). Together, these data reveal that bats have contended with retroviral infection throughout the course of their evolutionary history and suggest that retroviruses continue to circulate in bat populations today.

## ERVs PROVIDE HINTS TO OTHER XRVs HIDDEN IN THE WILD

ERVs provide a historical record of retroviral invasions of animal genomes. Beyond that, they provide clues to the existence of XRVs yet to be discovered. In the case of the two recent reports of bat XRVs (16, 17), ERV analysis preceded XRV discovery. A putative bat gammaretroviral ERV, flying fox retrovirus 1 (FFRV1), which is phylogenetically closely positioned to HPG (16), was reported a year earlier (82). Similarly, endogenous bat deltaretroviruses were reported (31) prior to the discovery of EfDRV (17). These two bat XRVs are from different retroviral genera, found in distantly related bats on opposite sides of the world (Fig. 2). It would be surprising if other species of bats are not hosting other diverse exogenous retroviruses. So, what clues does the ERV genomic fossil record hold? As the vast majority of bat genomes remain to be sequenced, many such clues remain to be found. However, the relatively small number of bat genomes reported thus far have already provided some interesting leads.

An analysis of endogenous bat betaretroviruses in 2013 revealed the first fully intact bat ERV, M. lucifugus endogenous betaretrovirus C (MlERV-βC), in the genome of the little brown bat (M. lucifugus, family Vespertilionidae) (30). MlERV-βC potentially represents a replication competent betaretrovirus since there is only minor divergence between its LTRs (integration ∼4.2 mya), and has no indels, frameshifts, or other mutations disrupting key betaretroviral sequence motifs (30). Analysis of another bat betaretrovirus, Desmodus rotundus endogenous betaretrovirus (DrERV), in the genome of the common vampire bat (D. rotundus, family Phyllostomidae) was reported as a member of a group of mammalian betaretroviruses involved in multiple cross-species transmission events, the most recent of which occurred ∼0.9 mya (65). Such recent integration suggests that this group may be represented among circulating XRVs.

Reports detailing endogenous bat gammaretroviruses also suggest the presence of yet-to-be-discovered XRVs (61, 79). Analysis of the M. lucifugus genome revealed a group of gammaretroviral ERVs, M. lucifugus endogenous retrovirus family 1 (MLERV1), that has additionally invaded the germ line of cats and pangolins (79). Close relatives of MLERV1 have undergone evolutionarily recent integrations, suggesting that they may still be circulating (79). The endogenous bat gammaretrovirus, RfRV, from the greater horseshoe bat (Rhinolophus ferrumequinum, family Rhinolophidae) was first reported as a defective retrovirus detected in the transcriptome of R. ferrumequinum (29). Later analysis failed to find evidence of RfRV in the genome of *R. ferrumequinum* or nine other bats (61). This suggests that RfRV may exist as a defective ERV in a subset of its host’s gene pool and is not yet fixed in the bat population. This, in turn, hints that XRV relatives of RfRV may yet be circulating in the wild.

The recent study detailing HPG also reported the presence of two closely related gammaretrovirus sequences, Macroglossus minimus gammaretrovirus (MmGRV) and Syconycteris australis gammaretrovirus (SaGRV), from metagenomic analysis of two Australian bats, the long-tongued nectar bat (M. minimus, family Pteropodidae) and the common blossom bat (S. australis, family Pteropodidae), respectively (16). Coverage of these genomes was almost complete with the exception of the terminal repeat and unique 5′ and 3′ LTR regions, and no obvious deleterious mutations were found. Combined with their close relatedness to the exogenous HPG, these findings provide further evidence for the existence of a group of infectious gammaretroviruses circulating in multiple species across diverse bat families.

## THE ROLE OF BATS IN THE CROSS-SPECIES TRANSMISSION OF RETROVIRUSES

The retroviral family consists of XRVs and ERVs from highly diverse animal species (83). Various broad phylogenetic analyses have revealed that while retroviruses may stably evolve and diverge with their host, cross-species transmission of retroviruses, including between different orders of mammals, is a common occurrence (30, 61, 66, 83–86).

Bat retroviruses are no exception to these cross-species transmission events. Endogenous bat gamma- and betaretroviruses, also frequently described in the literature as class I and class II ERVs, respectively, are widely distributed across the gamma- and betaretroviral phylogenies, and intermixed among retroviruses from other animal species (28, 30, 66). An analysis of thousands of ERVs across 60 mammalian genomes reveals six independent origins of bat gammaretroviruses (66). A separate analysis of thousands of ERVs across 69 mammalian genomes demonstrates that bats play a major role, alongside rodents, as hosts of mammalian retroviruses (61). Bats are also implicated in frequent cross-species transmission events and are particularly adept at receiving retroviruses from other mammals (61).

As the only mammals capable of flight, bats are of particular interest regarding the spread of mammalian viruses. Because flight allows bats to cross physical barriers impenetrable to other mammals, bats may provide a hypothetical transmission route between urban and rural park and garden environments separated by human infrastructure, forming a potential transmission link between various wild and domestic animal species. Bats can travel over large distances and can cross biogeographical boundaries, including extensive bodies of water. For example, the habitat of the black flying fox (*P. alecto*), the host of HPG, ranges across the oceanic faunal boundary called the Wallace line (87, 88) (Fig. 2). HPG is a close relative of KoRV and GALV, which are similarly closely related to each other (77.5% nucleotide identity) (16). The close relatedness of KoRV and GALV is intriguing since their hosts, koalas and gibbons, respectively, exist in nonoverlapping habitats in Australia and South East Asia, and separated by the Wallace line. For many years, bats have been suspected as intermediary hosts facilitating a transmission link between the environments and hosts of KoRV and GALV (66, 89–91). The identification of HPG as a close relative of KoRV, in an Australasian bat linking these habitats (16), supports this view.

## BATS AND THE ORIGINS OF KoRV-RELATED RETROVIRUSES

The specific role of bats in the transmission network of viruses closely related to KoRV and GALV across the Australasian region remains unclear, and many questions remain. Did this group of gammaretroviruses originate in Australia or Asia? Was transmission of the virus across the Wallace line a once-off, chance event, or are these viruses regularly carried across this barrier, perhaps in both directions? How many other mammalian host species are involved? Which mammals are natural reservoir hosts, and which represent incidental cross-species transmission events?

In addition to gibbons, koalas, and bats, rodents also play an important and closely connected role in this story. Two studies have reported the presence of GALV-like retroviruses in an Australasian rodent, the grassland melomys (Melomys burtoni). Importantly, these were from two subspecies of M. burton*i*, one from Australia (91), and one within Wallacea, Indonesia, on the east side of the Wallace line (92). The Wallacean GALV-like virus, *Melomys* woolly monkey virus (MelWMV), is an ERV with two large deletions in the *pol* and *env* genes (92). The Australian GALV-like virus, *M. burtoni* retrovirus (MbRV), was reported as four short sequences derived from the *pol* and *env* genes likely representing an ERV (91). MbRV and MelWMV are phylogenetically very closely related, both to each other, and to the woolly monkey virus (WMV) (92).

Intriguingly, the recently reported KoRV-related bat viruses do not form a single clade within the KoRV/GALV family tree (16). HPG and its closest relatives, which are hosted by several Australian pteropid bat species, form a clade that is basal to both KoRV and GALV, while the RhGRV and HlGRV viruses, which are hosted by Asian microbat species, form an intermediate clade between the WMV/MbRV and GALV clades (16). This phylogenetic analysis of KoRV-related viruses (16) indicates that we currently only have a handful of pieces of a very large puzzle, and it is unlikely that we can confidently determine the origin of HPG and KoRV until we fill in the gaps with closely related viruses in the phylogenetic tree. Specifically, KoRV is in a distinct clade to HPG and GALV, and the common ancestor of these viruses is unknown.

In the meantime, we might hypothesize that the KoRV/GALV group of gammaretroviruses originated in Australia, likely millions of years ago, perhaps in a bat, or rodent, or another mammal. One lineage of descendants is widely present in, and currently circulating among several species of Australian pteropid bats, some of which range up into South East Asia, and across the Wallace line (16). At some point after the divergence of the pteropid lineage, an Australian animal became infected with the direct ancestor of KoRV (93). KoRV is too dissimilar to HPG and other presently known retroviruses to conclude that any particular species is responsible for transmission of KoRV to koalas (16). Another descendant lineage has been circulating in native Australian melomys rodents, one variety of which presently inhabits South East Asia (91, 92). Spreading then to Asia, at least two species of Asian microbats and two Asian primate species became infected with viruses more closely related to the melomys than the pteropid lineage (16, 90). Some of these animals, such as the primates, likely represent incidental hosts since these were very recent (on the scale of decades) cross-species transmission events. Investigations of the origin of GALV point to an iatrogenic infection of gibbons in a South East Asian medical research facility, and GALV has even been hypothesized to have originated as a reverse zoonosis from an as yet undiscovered human gammaretrovirus in Papua New Guinea (94, 95). Additional sampling of rodents, marsupials, bats, and other species will be required to reveal the events leading to the emergence of KoRV and the spread of KoRV-related viruses across Australasia.

## A THREAT WAITING IN THE WINGS? IS THERE POTENTIAL FOR HUMAN INFECTION?

The ultimate question is “Are bat retroviruses a threat to humans?” What does the evidence allow us to speculate at this point? We know that retroviruses can infect humans as exemplified by HIV and HTLV viruses that have caused significant morbidity and mortality (10–12). Our ancestors were certainly infected by other retroviruses, as evidenced by the fossil record of beta- and gammaretroviral ERVs in our genome, some of which likely integrated within the last million years (96–98). Among mammals, interorder cross-species transmission is a common occurrence for retroviruses (66).

The recently reported bat XRVs are delta- and gammaretroviruses (16, 17). HTLV is a deltaretrovirus, and modern gammaretroviral XRVs (e.g., GALV) are known to infect nonhuman primates (95). That said, there is currently no credible evidence that gammaretroviruses can infect and cause disease in humans. While we should remain cautiously skeptical about the potential for novel retroviral infections of humans, there is no fundamental reason to believe that bat retroviruses cannot infect humans. Accordingly, speculation around this question will focus on specific features affecting potential infection, such as host cell permissiveness to specific XRVs.

While no infectivity analyses have been reported for EfDRV, we can consider its potential to infect humans by looking at its closest exogenous relatives, HTLV and BLV. BLV uses the CAT1 (SLC7A1) receptor during cell entry and is capable of replication in human cells *in vitro*, and nonpermissive cells become permissive when ectopically expressing human CAT1 (99, 100). While controversial, several lines of evidence have emerged in recent years suggesting a connection between BLV and human breast cancer (101, 102). It is important to note that while multiple groups have reported significant associations between the presence of BLV DNA in human breast cancers (103–105), multiple other groups have found no association (106–108). The potential link between BLV and human breast cancer requires further cautious analysis, and confirmation of the integration of BLV into the genome of human cancer cells, which is necessary for productive infection, and the expression of viral proteins associated with oncogenesis, remains to be demonstrated. EfDRV is even more closely related to HTLV than BLV based on a limited phylogenetic analysis of the Gag protein sequence (17). HTLV is a well-known human pathogen, and combined with what has been recently reported for BLV, it is not unreasonable to speculate that the bat retrovirus EfDRV is among a clade of deltaretroviruses with shared potential for human infection.

HPG is closely related to the XRVs, KoRV-A and GALV, both of which use the PiT-1 (SLC20A1) receptor for cell entry (90, 109, 110). HPG displays a similar pattern of cell tropism as KoRV-A and GALV, and cells persistently infected by HPG are resistant to superinfection by KoRV-A and GALV (16). This indicates that HPG also uses the PiT-1 receptor, which is unsurprising given the close relatedness between these viruses. PiT-1 is ubiquitously expressed across mammalian cells (111–113), and humans share the same permissivity motif within PiT-1 enabling interaction with KoRV-related viral envelope proteins (16). Furthermore, HPG is capable of infecting human cells *in vitro*, and viral particles released by these cells are further capable of establishing persistent infections in human cells (16). GALV is closely related to HPG, and it has been well documented that GALV causes infection of nonhuman primates (95) and is capable of replication in human cells (114).

HPG can infect human cells *in vitro*; however, this does not necessarily equate to the virus being able to establish infection and cause disease in humans. Here we should consider the cautionary tale of the xenotropic murine leukemia virus-related virus (XMRV), incorrectly identified as a human gammaretrovirus associated with prostate cancer and chronic fatigue syndrome (115–117). XMRV is capable of infecting human cells *in vitro* (118). However, in nonhuman primate infection studies, it established only a limited infection with no pathological consequences (119–121), and its role in infecting and causing disease in humans was disproven (115, 117, 122–125). Other discoveries of retroviruses have initially been linked to human diseases, only to be later disproved, to the extent that the moniker “human rumour viruses” has been applied to them (126). The history of retroviral “false-positive” results linked to human diseases ensures that overwhelming evidence will be required for new retroviral etiologies of human infectious diseases to be accepted.

Interspecies transmission of retroviruses could occur through a number of scenarios. For example, GALV and KoRV have been detected in various body tissues and fluids, including feces, urine, blood, breast milk, and sperm (57, 93, 109, 127–131). Accordingly, it is possible that transmission could occur through routes, including the contamination of food sources by feces or urine, or through blood during fighting/predation. With specific respect to the potential for transmission between bats and humans, various conditions must be met for spillover of virus to occur (132), but the use of bats as a source of bushmeat and the ever-expanding encroachment of humans into bat habitats (1, 133) would seem to provide no shortage of instances with potential for bat-to-human transmission of viruses, including potentially retroviruses.

## CONCLUSION AND OUTLOOK

Bats have long contended with retroviral infections, and new research demonstrates that this is still the case, with two distantly related infectious retroviruses infecting two distantly related bats in different corners of the world. Clues from the genomic fossil record and the antiretroviral defenses within the bat immune system suggest that there may be many as yet undiscovered bat retroviruses circulating in the wild. The frequency of retroviral transmission between mammalian species, the evolutionarily important role that bats have played in these cross-species transmission events, and the capacity for biological compatibility between human cells and bat retroviruses may be collectively read as a warning that we should not dismiss the potential for transmission of retroviruses from bats to humans, potentially via intermediate hosts.

So where to from here? Moving forward, we will need to consider the challenges involved in unlocking the secrets of bats and their viruses, such as the lack of reagents to study bats and the difficulty involved in isolating viruses (2, 32). Continued surveillance of bats for the presence of novel retroviruses and subsequent investigations of the biological characteristics and infectious potential held by such viruses will facilitate a proactive approach in the event of a zoonotic spillover involving a bat retrovirus. Given the inherent difficulties in distinguishing ERVs and XRVs, it is possible that some reported bat retrovirus sequences have, and will, actually represent XRVs, but closer analysis than is often applied is required to shed light in either direction. High thresholds of evidence will be required for ongoing identification of infectious bat retroviruses, a bar that is perhaps too high for many broad viral metagenomic studies, but which should be embraced if we are to uncover the full picture of the potential zoonotic threats harbored by bats.
